# Supplementary material for: Enhanced plasma half-life and efficacy of engineered human albumin-fused GLP-1 despite enzymatic cleavage of its C-terminal end
Source: Commun Biol. 2025 May 26;8:810. doi: 10.1038/s42003-025-08249-8 (PMC12106674; doi:10.1038/s42003-025-08249-8)
Supplement: Supplementary file 2 — Supplementary Information [file 42003_2025_8249_MOESM2_ESM.pdf]

SUPPLEMENTARY INFORMATION FOR

Enhanced plasma half-life and efficacy of engineered albumin-fused GLP-1 despite enzymatic cleavage of its C-terminal end

Jeannette Nilsen, Kristin Hovden Aaen, Sopisa Benjakul, Fulgencio Ruso Julve, Thomas Uwe Greiner, Daniela Bejan, Maria Stensland, Sachin Singh, Tilman Schlothauer, Inger Sandlie, and Jan Terje Andersen[#].

#Corresponding author: Email: j.t.andersen@medisin.uio.no (J.T.A.).

Contents of supplementary information:

Supplementary tables

Supplementary Table 1. C-terminal peptides of 585-engineered albumin variants upon exposure to CPA

Supplementary Table 2. C-terminal peptides of WT and QMP albumin, and GLP-1, FIX, and ACE2 fused to WT albumin or QMP albumin upon exposure, or no exposure, to CPA

Supplementary Table 3. Theoretical pI and net charge of unfused albumin variants and GLP-1 albumin fusions

Supplementary Table 4. Non-compartmental analysis of PK studies in human FcRn Tg32 mice

Supplementary figures

Supplementary Figure 1. Production yield of unfused albumin variants and GLP-1 albumin fusions

Supplementary Figure 2. FcRn binding kinetics of 585-engineered albumin variants

Supplementary Figure 3. FcRn binding kinetics of unfused albumin variants and GLP-1 albumin fusions

Supplementary Figure 4. Thermal stability analysis of unfused albumin variants and GLP-1 albumin fusions

Supplementary Figure 5. pH-dependent net charge of unfused albumin variants and GLP-1 albumin fusions

Supplementary Figure 6.	Cellular uptake and recycling of unfused albumin variants and GLP-1 albumin fusions
Supplementary Figure 7.	Effect of enzymatic cleavage by CPA on FcRn binding on albumin fusions
Supplementary Figure 8.	Plasma concentrations of Tanzeum and GLP-1 WT in human FcRn Tg32 mice
Supplementary Figure 9.	SDS-PAGE analysis of unfused albumin variants and GLP-1 albumin fusions

Supplementary Table 1. C-terminal peptides of 585-engineered albumin variants upon exposure to CPA.

C-terminal peptide	WT		L585I		L585M		L585V	
	(-)[a]	(+)[b]	(-)	(+)	(-)	(+)	(-)	(+)
LVAASQAALG-	3.78×10^6	6.43×10^9	6.29×10^6	7.73×10^9	4.59×10^6	8.03×10^9	5.55×10^6	8.24×10^9
KLVAASQAALG-	ND[c]	7.50×10^8	ND	1.40×10^9	ND	1.06×10^9	ND	1.05×10^9
LVAASQAALGL	9.96×10^9	2.00×10^7	–	–	ND	ND	1.08×10^7	ND
KLVAASQAALGL	6.65×10^8	ND	–	–	ND	ND	ND	ND
LVAASQAALGI	–	–	1.26×10^{10}	2.00×10^8	–	–	–	–
KLVAASQAALGI	–	–	ND	ND	–	–	–	–
LVAASQAALGM	ND	ND	ND	ND	1.12×10^{10}	1.44×10^9	ND	ND
KLVAASQAALGM	ND	ND	ND	ND	6.11×10^8	4.53×10^7	ND	ND
LVAASQAALGV	ND	ND	ND	ND	ND	ND	2.73×10^7	1.18×10^9
KLVAASQAALGV	ND	ND	ND	ND	ND	ND	1.86×10^9	1.19×10^8
**Percentage of peptides lacking L585 (%)**	**0.04**	**99.72**	**0.05**	**98.64**	**0.04**	**85.92**	**0.29**	**87.71**

[a] (-), Without CPA exposure.
[b] (+), With CPA exposure.
[c] ND, Not detected.

Supplementary Table 2. C-terminal peptides of WT albumin and QMP, and GLP-1, FIX, and ACE2 fused to WT albumin or QMP upon exposure, or no exposure, to CPA.

**C-terminal peptide**		**KLVAASQAALG-**	**LVAASQAALG-**	**KLVAASQAALGL**	**LVAASQAALGL**	**Percentage of peptides lacking L585 (%)**
WT	(-)[a]	–[c]	–	–	4.41×10^{10}	0.00
	(+)[b]	2.86×10^{9}	2.0×10^{10}	–	–	100.00
QMP	(-)	–	–	–	8.25×10^{8}	0.00
	(+)	–	2.58×10^{10}	–	–	100.00
GLP-1 WT	(-)	–	–	3.42×10^{7}	6.53×10^{10}	0.00
	(+)	2.07×10^{9}	2.23×10^{10}	–	–	100.00
GLP-1 QMP	(-)	–	–	–	5.96×10^{10}	0.00
	(+)	–	2.01×10^{10}	–	–	100.00
ACE2 WT	(-)	–	–	–	–	0.00
	(+)	5.98×10^{8}	7.40×10^{9}	–	–	100.00
ACE2 QMP	(-)	–	–	–	–	0.00
	(+)	–	4.77×10^{9}	–	–	100.00
FIX WT	(-)	–	–	–	2.76×10^{9}	0.00
	(+)	1.67×10^{9}	1.95×10^{10}	–	–	100.00
FIX QMP	(-)	–	–	–	6.53×10^{8}	0.00
	(+)	–	1.75×10^{10}	–	–	100.00

[a](-), Without CPA exposure.
[b](+), With CPA exposure.
[c]–, Not detected.

Supplementary Table 3. Theoretical pI and net charge of unfused albumin variants and GLP-1 albumin fusions.

Variant	pI[a]	Net charge	
		pH 5.5	pH 7.4
WT	5.8	+2.8	–14.7
QMP			
QMP/LX			
$(GLP\text{-}1\ (7–36))_2$	5.6	+0.1	–2.22
GLP-1 WT	5.8	+2.9	–15.5
GLP-1 LX			
GLP-1 QMP			
GLP-1 QMP/LX			

[a] pI, isoelectric point.

Supplementary Table 4. Non-compartmental analysis of PK studies in human FcRn Tg32 mice.

Variant	Model	Dose (mg/kg)	Route	AUC [e] (µg·d/mL)	C_{max} [f] (µg/mL)	CL [g] (mL/kg/d)	MRT [h] (d)	V_{ss} [i] (mL/kg)	$T_{1/2}$ [j] (d)	R^2 [k]
WT	Hemi[a]	1	IV	5.8	1.5	155.3	3.5	540.0	2.5	0.979
QMP	Hemi	1	IV	21.8	3.2	40.6	8.3	333.0	7.3	0.985
QMP/LX	Hemi	1	IV	19.1	2.9	46.6	8.1	376.5	7.4	0.990
Tanzeum	Homo[b]	4	IV	49.9	14.2	78.2	2.3	178.2	1.7	1.000
GLP-1 WT [c]	Homo	4	IV	58.0	17.2	67.6	2.2	150.2	1.6	0.999
GLP-1 WT [d]	Homo	4	IV	58.4	17.2	67.1	1.9	126.3	1.7	0.995
GLP-1 LX	Homo	4	IV	42.3	14.0	93.3	1.2	113.8	1.2	0.986
GLP-1 QMP	Homo	4	IV	156.2	32.0	22.7	4.9	110.2	3.9	0.996
GLP-1 QMP/LX	Homo	4	IV	148.1	27.8	23.4	5.3	125.1	3.9	1.000
GLP-1 WT	Homo	4	SC	56.7	18.0	68.4	2.7	-	1.8	0.973
GLP-1 QMP	Homo	4	SC	176.0	26.4	19.5	6.5	-	3.8	0.986

[a] Hemi, Hemizygous human FcRn Tg32 mice.
[b] Homo, Homozygous human FcRn Tg32 mice.
[c] GLP-1 WT in an experiment compared side-by-side with Tanzeum.
[d] GLP-1 WT in an experiment compared side-by-side with GLP-1 LX, GLP-1 QMP, and GLP-1 QMP/LX.
[e] AUC, Area under the curve from time 0 to the last measured time point.
[f] C_{max}, Maximum plasma concentration, achieved at 24 hours for all groups.
[g] CL, Clearance rate.
[h] MRT, Mean residence time.
[i] V_{ss}, Volume of distribution at steady-state.
[j] $T_{1/2}$, Terminal half-life.
[k] R^2, Coefficient of determination.

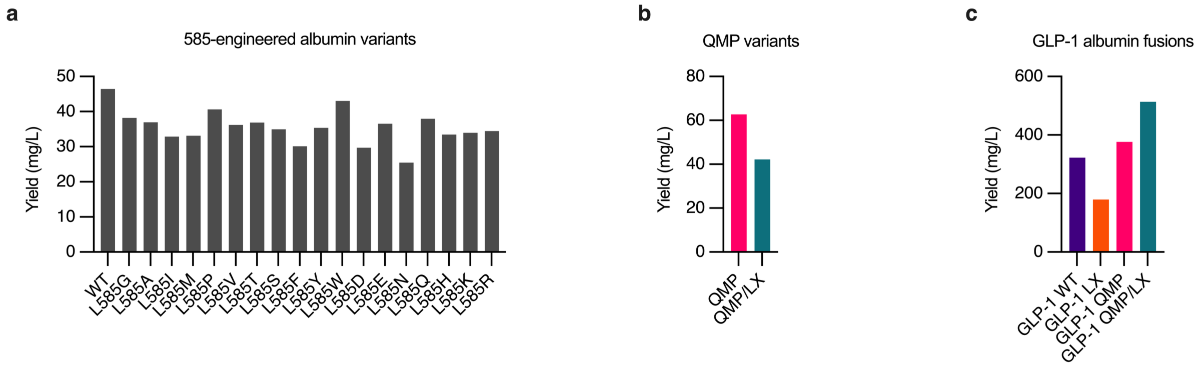

Supplementary Figure 1. Production yield of unfused albumin variants and GLP-1 albumin fusions. The production yield of purified fractions of recombinant **a,** full-length WT albumin and 585-engineered albumin variants, **b,** QMP variants, and **c,** GLP-1 albumin fusions, produced in Expi293F cells.

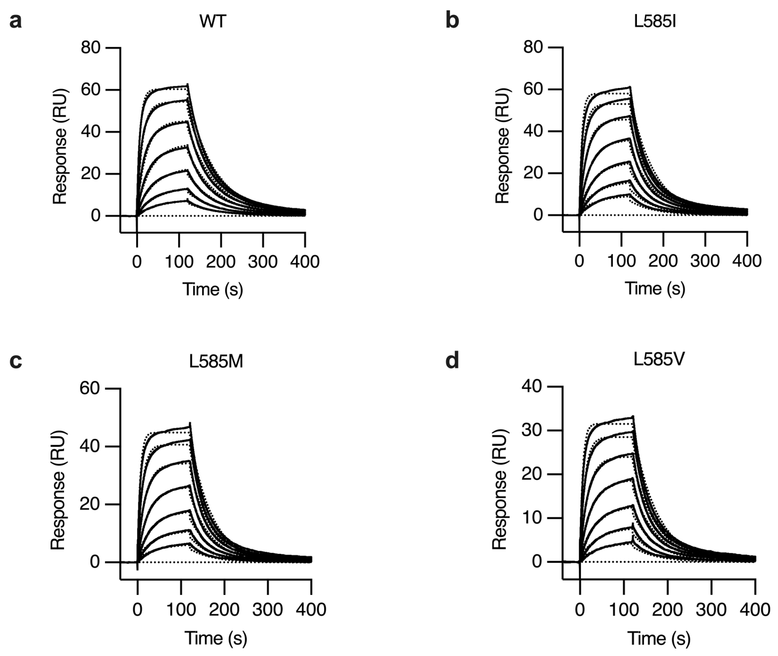

Supplementary Figure 2. FcRn binding kinetics of 585-engineered albumin variants. Representative SPR sensorgrams showing the binding of serial dilutions (78 nM – 5000 nM) of **a,** WT albumin, **b,** L585I, **c,** L585M, and **d,** L585V injected over immobilized biotinylated human FcRn at pH 5.5 (solid lines). The albumin variants were injected in duplicates. The data were fitted to the 1:1 Langmuir binding model (dotted lines).

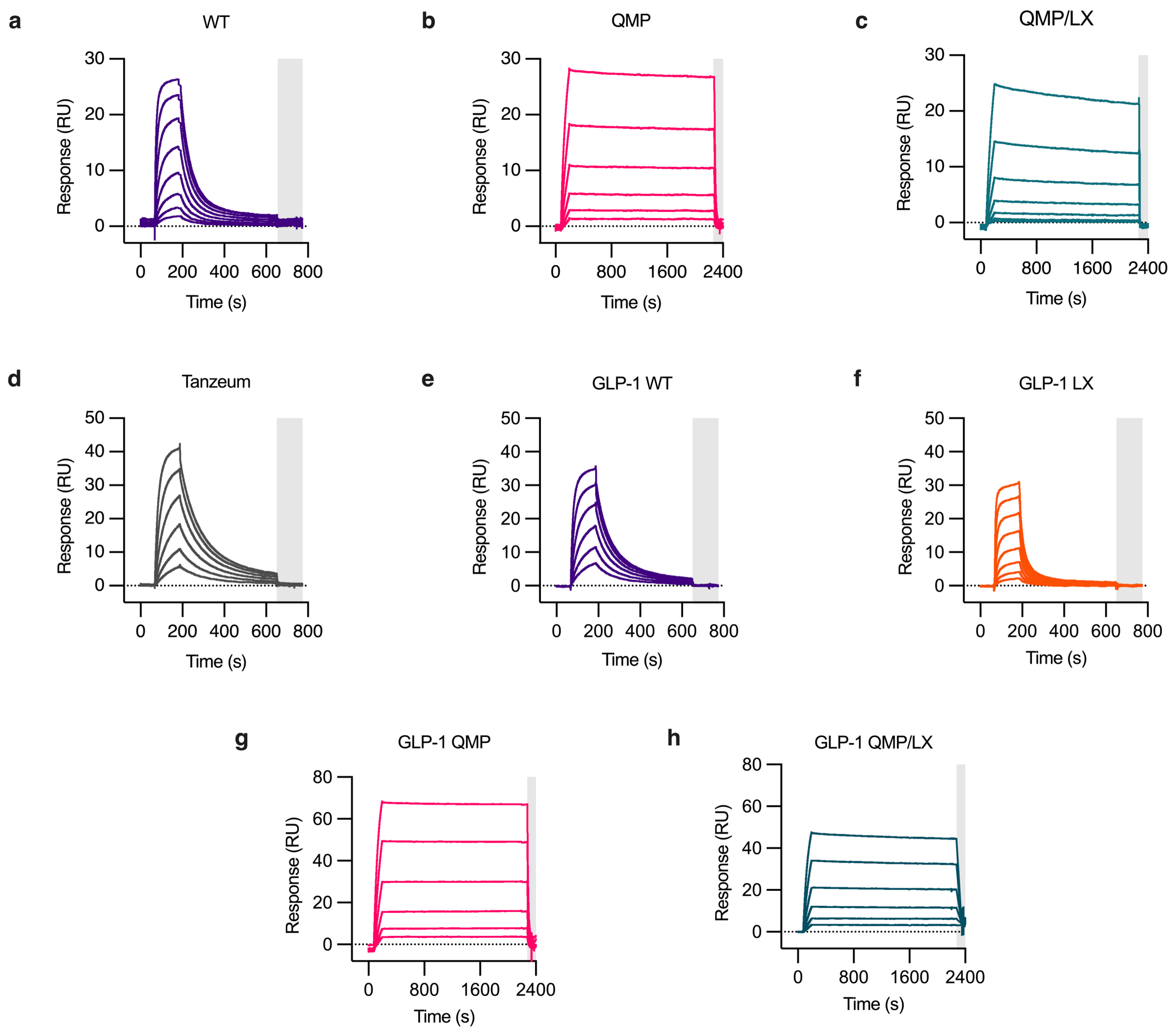

Supplementary Figure 3. FcRn binding kinetics of unfused albumin variants and GLP-1 albumin fusions. Representative SPR sensorgrams showing the binding of serial dilutions of **a–c,** unfused albumin variants and **d–h,** GLP-1 albumin fusions. (a) WT albumin (39 nM – 5000 nM), (b) QMP (1.6 nM – 50 nM), (c) QMP/LX (1.6 nM – 50 nM), (d) Tanzeum (19.5 nM – 625 nM), (e) GLP-1 WT (19.5 nM – 1250 nM), (f) GLP-1 LX (39 nM – 5000 nM), (g) GLP-1 QMP (1.6 nM – 50 nM), and (h) GLP-1 QMP/LX (1.6 nM – 50 nM) were injected in duplicates over immobilized biotinylated human FcRn at pH 5.5. Regeneration of the sensor chip surface was performed by injection of PBS (pH 7.4) at the time points indicated by the grey areas.

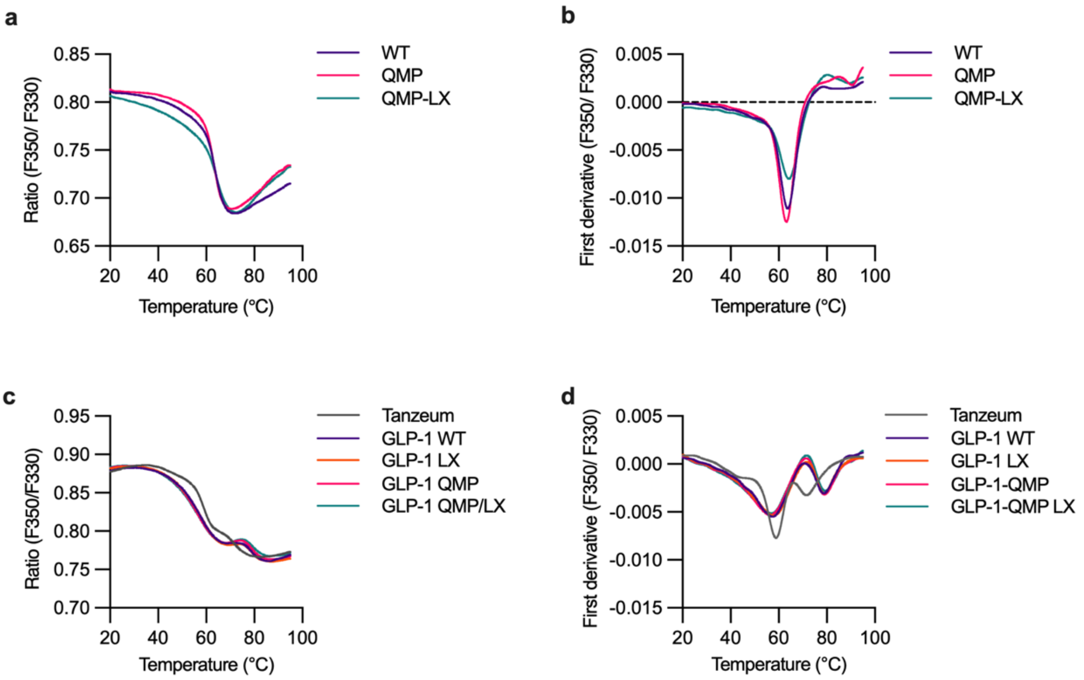

Supplementary Figure 4. Thermal stability of unfused albumin variants and GLP-1 albumin fusions. Thermal unfolding curves of **a and b,** unfused albumin variants and **c and d,** GLP-1 albumin fusions in PBS (pH 7.4), shown as the ratio of fluorescence intensities at 350 nm and 330 nm, or the ratio first derivate, as a function of temperature. The values represent the mean from one representative experiment (*n* = 2).

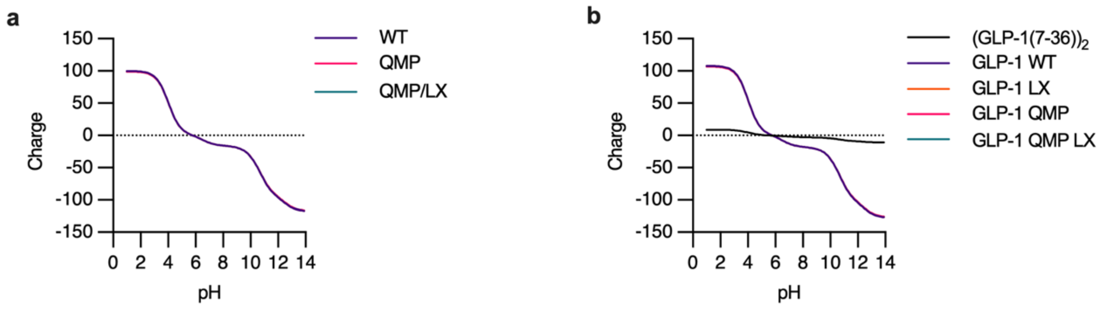

Supplementary Figure 5. pH-dependent net charge of unfused albumin variants and GLP-1 albumin fusions. Theoretical net charge of **a,** unfused albumin variants and **b,** unfused GLP-1 or GLP-1 albumin fusions, as a function of pH (pH 1 to pH 13.9), calculated with EMBOSS iep.

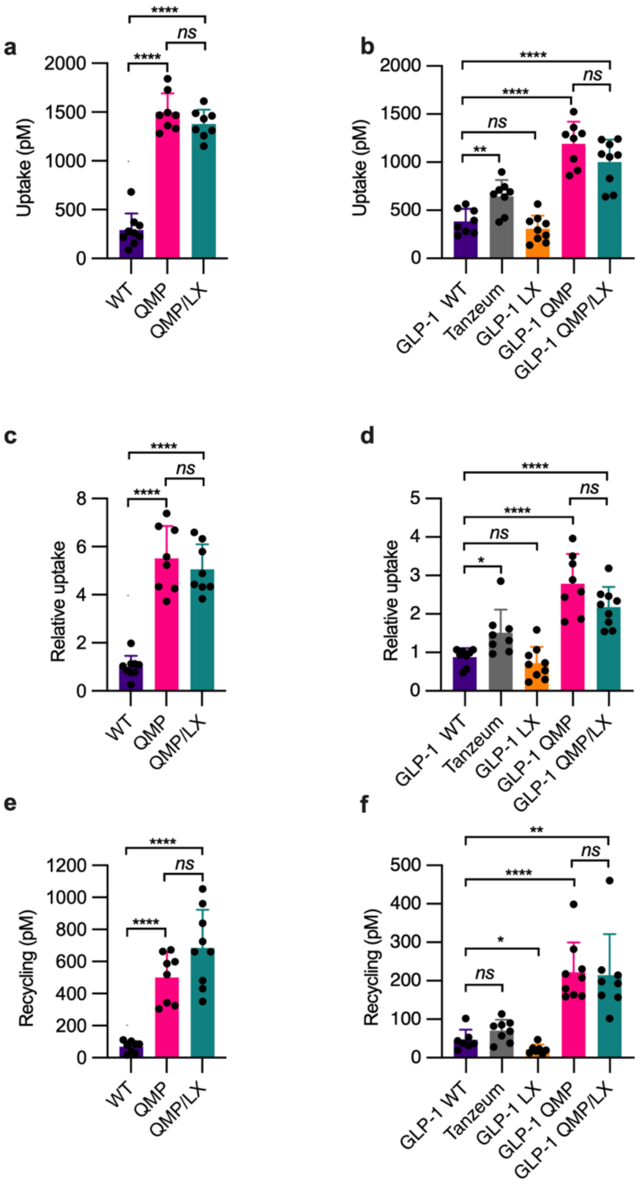

Supplementary Figure 6. Cellular uptake and recycling of unfused albumin variants and GLP-1 albumin fusions. HERA results showing the amount of **a,** unfused albumin variants and **b,** GLP-1 albumin fusions inside the HMEC-1-FcRn cells following the uptake phase, and relative uptake values for the **c,** unfused albumin variants and **d,** GLP-1 albumin fusions, where WT albumin or GLP-1 WT were set to 1 in each independent experiment. HERA results showing the amount of recycled **e,** unfused albumin variants and **f,** GLP-1 albumin fusions released into the media. The values represent the mean ± SD from three independent experiments (n = 3). Unpaired two-tailed t-tests were used for statistical analysis, where *ns* = not significant, $*p < 0.05$, $**p < 0.005$, $***p < 0.0005$, and $****p < 0.0001$.

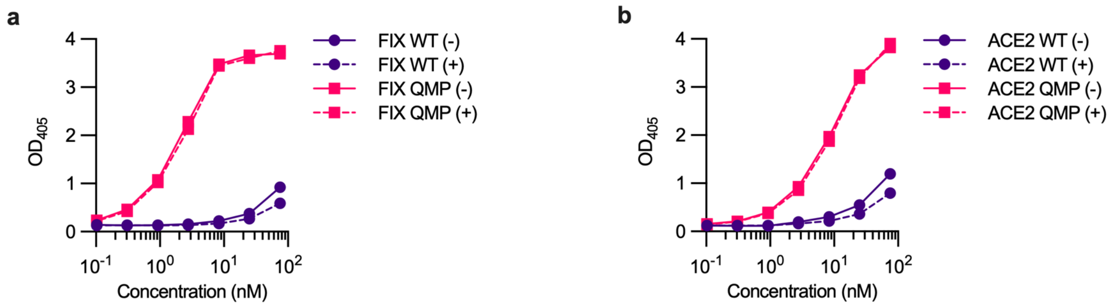

Supplementary Figure 7. Effect of enzymatic cleavage by CPA on FcRn binding of albumin fusions. ELISA results showing the binding of WT albumin and QMP fused **a,** FIX and **b,** ACE2, without (-) and with (+) exposure to CPA, to human FcRn at pH 5.5. The values represent the mean from one representative experiment (n = 2).

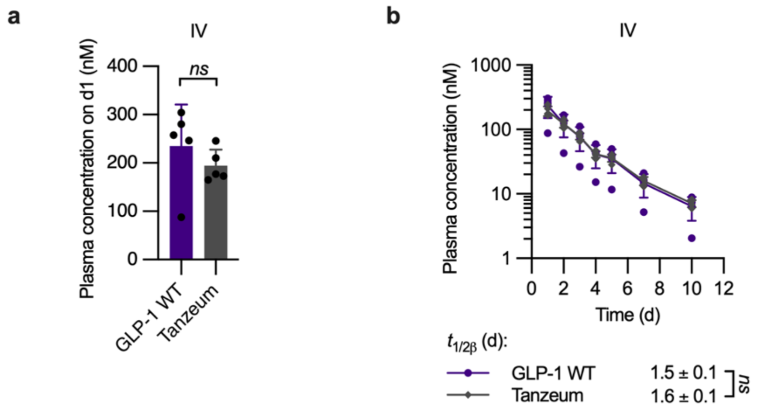

Supplementary Figure 8. Plasma concentrations of Tanzeum and GLP-1 WT in human FcRn Tg32 mice. Plasma concentrations of GLP-1 WT (purple), and Tanzeum (grey) **a,** on day 1 and **b,** from day 1 to day 10 following IV administration to homozygous human FcRn Tg32 mice. The values represent the mean ± SD from one representative experiment (*n* = 5). The plasma half-lives ($t_{1/2\beta}$) are shown as the mean ± SD. Unpaired two-tailed *t*-tests were used for statistical analysis, where *ns* = not significant.

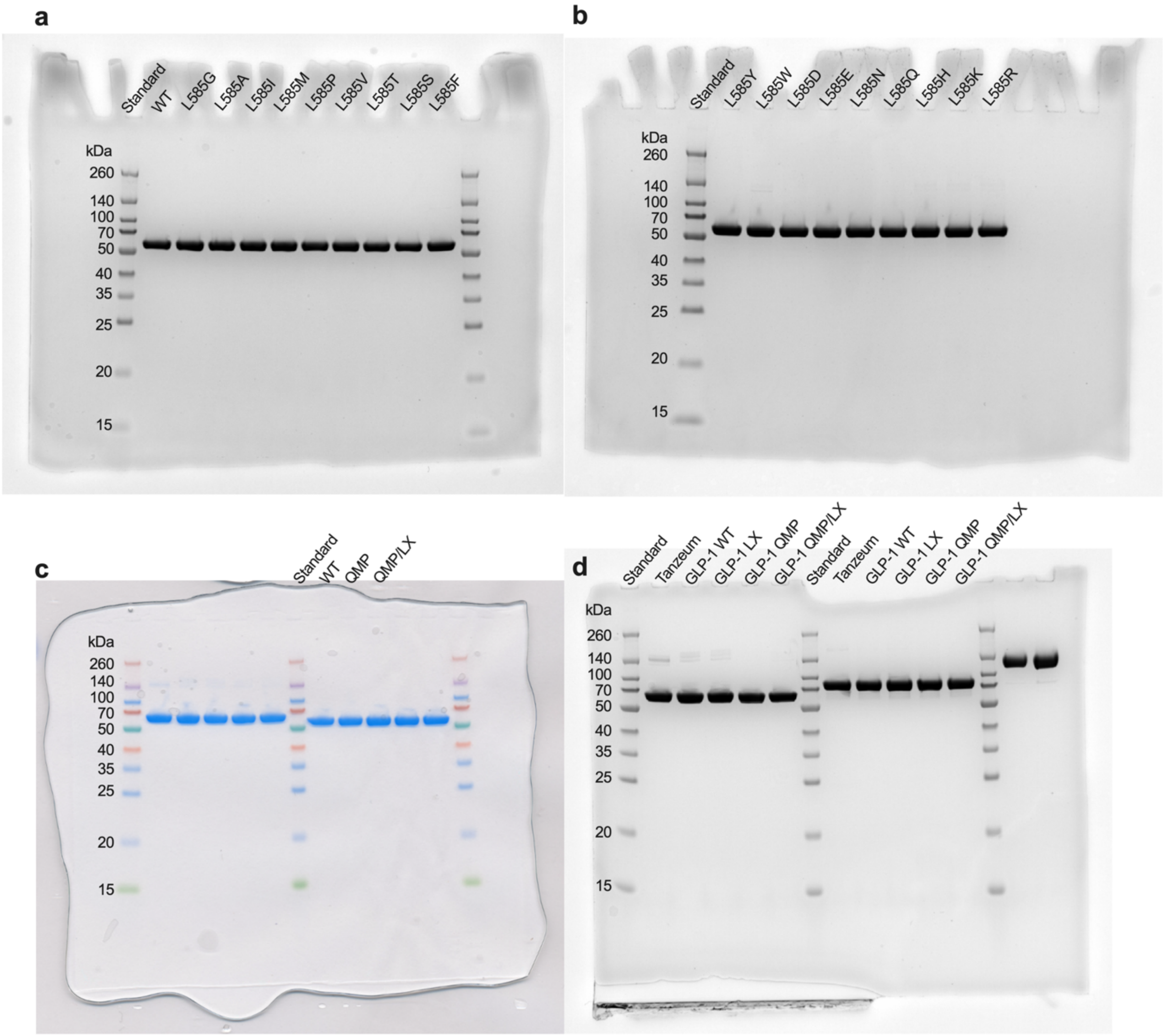

Supplementary Figure 9. SDS-PAGE gels of unfused albumin variants and GLP-1 albumin fusions. 12% SDS-PAGE gels stained with Coomassie brilliant blue showing purified fractions of **a–c,** recombinant full-length WT albumin and human albumin variants (66.5 kDa) produced in Expi293F cells, under non-reducing conditions, and **d**, Tanzeum and GLP-1 albumin fusions (73 kDa) produced in Expi293F cells, under non-reducing and reducing conditions.
