# Supplementary material for: Enhanced plasma half-life and efficacy of engineered human albumin-fused GLP-1 despite enzymatic cleavage of its C-terminal end
Source: Commun Biol. 2025 May 26;8:810. doi: 10.1038/s42003-025-08249-8 (PMC12106674; doi:10.1038/s42003-025-08249-8)
Supplement: Supplementary file 3 — Description of Additional Supplementary Materials [file 42003_2025_8249_MOESM3_ESM.pdf]

Description of Additional Supplementary Files

File name: Supplementary Data 1

Description: Source data underlying graphs presented in the main figures
