# Supplementary material for: Enhanced plasma half-life and efficacy of engineered human albumin-fused GLP-1 despite enzymatic cleavage of its C-terminal end
Source: Commun Biol. 2025 May 26;8:810. doi: 10.1038/s42003-025-08249-8 (PMC12106674; doi:10.1038/s42003-025-08249-8)
Supplement: Supplementary file 5 — Reporting Summary [file 42003_2025_8249_MOESM5_ESM.pdf]

Corresponding author(s): Jan Terje Andersen
Last updated by author(s): May 9, 2025

Reporting Summary

Nature Portfolio wishes to improve the reproducibility of the work that we publish. This form provides structure for consistency and transparency in reporting. For further information on Nature Portfolio policies, see our Editorial Policies and the Editorial Policy Checklist.

Statistics

For all statistical analyses, confirm that the following items are present in the figure legend, table legend, main text, or Methods section.

n/a	Confirmed	
☐	☒	The exact sample size (*n*) for each experimental group/condition, given as a discrete number and unit of measurement
☒	☐	A statement on whether measurements were taken from distinct samples or whether the same sample was measured repeatedly
☐	☒	The statistical test(s) used AND whether they are one- or two-sided<br>*Only common tests should be described solely by name; describe more complex techniques in the Methods section.*
☒	☐	A description of all covariates tested
☒	☐	A description of any assumptions or corrections, such as tests of normality and adjustment for multiple comparisons
☐	☒	A full description of the statistical parameters including central tendency (e.g. means) or other basic estimates (e.g. regression coefficient) AND variation (e.g. standard deviation) or associated estimates of uncertainty (e.g. confidence intervals)
☐	☒	For null hypothesis testing, the test statistic (e.g. *F*, *t*, *r*) with confidence intervals, effect sizes, degrees of freedom and *P* value noted<br>*Give P values as exact values whenever suitable.*
☒	☐	For Bayesian analysis, information on the choice of priors and Markov chain Monte Carlo settings
☒	☐	For hierarchical and complex designs, identification of the appropriate level for tests and full reporting of outcomes
☒	☐	Estimates of effect sizes (e.g. Cohen's *d*, Pearson's *r*), indicating how they were calculated

Our web collection on statistics for biologists contains articles on many of the points above.

Software and code

Policy information about availability of computer code

Data collection

- Microsoft Excel; Microsoft 365 MSO; Version 2308 Build 16.0.16731.20542
- Protein Data Bank (PDB); https://www.rcsb.org/
- Expasy Translate; https://web.expasy.org/translate/
- Expasy ProtParam tool; https://web.expasy.org/protparam/
- Magellan; Tecan Biosciences; version 7.2
- Unicorn; Cytiva; version 7.1
- GraphPad Prism; GraphPad Software LLC; version 9.5.1
- BIAevaluation software; Cytiva; version 4.1
- PyMOL; PyMOL by Schrödinger; version 4.6.0
- MatLab; Mathworks; version R2021b
- gPKPDsim Toolbox; Mathworks; Version 1.1.3 (doi: 10.1007/s10928-017-9562-9)
- SimBiology; Mathworks; Version R2024a@
- EMBOSS iep software; https://www.bioinformatics.nl/cgi-bin/emboss/iep?_pref_hide_optional=0
- Perkin Elmer Victor3 1420 Multilabel Counter (Wallac 1420 Workstation, version 3.00 revision 5)

Data analysis

- Microsoft Excel; Microsoft 365 MSO; version 2308 Build 16.0.16731.20542
- GraphPad Prism; GraphPad Software LLC; version 9.5.1
- BIAevaluation software; Cytiva; version 4.1
- PyMOL; PyMOL by Schrödinger; version 4.6.0
- MatLab; Mathworks; version R2021b

- gPKPDsim Toolbox; Mathworks; Version 1.1.3 (doi: 10.1007/s10928-017-9562-9)
- SimBiology; Mathworks; Version R2024a

For manuscripts utilizing custom algorithms or software that are central to the research but not yet described in published literature, software must be made available to editors and reviewers. We strongly encourage code deposition in a community repository (e.g. GitHub). See the Nature Portfolio guidelines for submitting code & software for further information.

Data

Policy information about availability of data

All manuscripts must include a data availability statement. This statement should provide the following information, where applicable:

- Accession codes, unique identifiers, or web links for publicly available datasets
- A description of any restrictions on data availability
- For clinical datasets or third party data, please ensure that the statement adheres to our policy

Source data underlying graphs presented in the main figures can be found in Supplementary Data 1 and are available from the corresponding author upon reasonable request. The mass spectrometry proteomics data have been deposited to the ProteomeXchange Consortium (http://proteomecentral.proteomexchange.org) via the PRIDE partner repository with the dataset identifier PXD063561.

Publicly available data sets used:
Protein database accession number 6WUW.
Protein database accession number 4N0F.

Research involving human participants, their data, or biological material

Policy information about studies with human participants or human data. See also policy information about sex, gender (identity/presentation), and sexual orientation and race, ethnicity and racism.

Reporting on sex and gender	Not applicable, the study did not involve human participants, their data, or biological material.
Reporting on race, ethnicity, or other socially relevant groupings	Not applicable, the study did not involve human participants, their data, or biological material.
Population characteristics	Not applicable, the study did not involve human participants, their data, or biological material.
Recruitment	Not applicable, the study did not involve human participants, their data, or biological material.
Ethics oversight	Not applicable, the study did not involve human participants, their data, or biological material.

Note that full information on the approval of the study protocol must also be provided in the manuscript.

Field-specific reporting

Please select the one below that is the best fit for your research. If you are not sure, read the appropriate sections before making your selection.

☒ Life sciences ☐ Behavioural & social sciences ☐ Ecological, evolutionary & environmental sciences

For a reference copy of the document with all sections, see nature.com/documents/nr-reporting-summary-flat.pdf

Life sciences study design

All studies must disclose on these points even when the disclosure is negative.

Sample size

Sample size is in this setting relevant to mainly 1) animal experiments and 2) cellular experiments (HERA, GLP-1R reporter assay, GSIS assay).

Sample size in animal experiments was determined on the basis of ethical perspectives (the three Rs) and previously published data (see list 1) in the transgenic mouse strains, confirming the sufficiency of the used sample size for statistical interpretation.

Sample size in cellular experiments (HERA, GSIS assay) was determined on the basis of previously published data (see list 2) regarding method variability, the inclusion of technical replicates across biological replicates and necessary sample size for statistical interpretation where indicated.

For the GLP-1R reporter assay kit experiments, the sample size was chosen according to the manufacturer's recommendation.

1) Animal experiments: PMID: 35982144, PMID: 34109608, PMID: 32313072, PMID: 35118359, PMID: 15331566.
2) Cellular experiments: PMID: 29434196, PMID: 35982144, PMID: 37827516.

Data exclusions

Data was excluded from further interpretation if found to be below the sensitivity of the relevant analytic method, and has no impact on the inferred conclusions where applicable.

Outliers were identified using the ROUT method (Q= 1% or 2%) in GraphPad Prism and excluded.

Replication	To ensure reproducibility of experimental findings, analysis of plasma samples was performed at least twice, and the resulting data reviewed by internal experts.<br><br>Cellular experiments: HERA were performed at least three times and included at least three technical replicates of each individual data point. GLP-1R reporter assay was performed twice with three replicates.<br>GSIS assay was performed once with one to three technical replicates of each biological replicate.<br><br>Biochemical experiments were repeated at least once, analyzed using standard models within appropriate evaluation software and interpreted by at least two experienced individuals separately, in addition to being reviewed by external experts. All stated hardware underwent routinely maintenance and quality assessment both prior to and during the data collection period.<br><br>Modelling and structural analyses were performed by publically available methods and reviewed to fall within the expected standards of such analyses. Where applicable, custom software is made available to the reader for own review and reproduction.<br>All attempts at replication were successful.
Randomization	PK studies: Mice were randomly allocated into groups.<br>GTT studies: After the fasting period, blood glucose levels were measured, before mice of similar baseline values were evenly allocated between the groups.
Blinding	Irrelevant to our study, as it would compromise ability to interpret data.

Reporting for specific materials, systems and methods

We require information from authors about some types of materials, experimental systems and methods used in many studies. Here, indicate whether each material, system or method listed is relevant to your study. If you are not sure if a list item applies to your research, read the appropriate section before selecting a response.

Materials & experimental systems

n/a	Involved in the study	
☐	☒	Antibodies
☐	☒	Eukaryotic cell lines
☒	☐	Palaeontology and archaeology
☐	☒	Animals and other organisms
☒	☐	Clinical data
☒	☐	Dual use research of concern
☒	☐	Plants

Methods

n/a	Involved in the study	
☒	☐	ChIP-seq
☒	☐	Flow cytometry
☒	☐	MRI-based neuroimaging

Antibodies

Antibodies used	Commercial antibodies used in experimental analysis (Tradename, Supplier, Reference, Lot number):<br>1) Polyclonal anti-human albumin from goat, Sigma-Aldrich, A1151-1VL, SLCF5233<br>2) ALP-conjugated anti-human albumin from goat, Bethyl Laboratories, Inc., A80-229AP, 10<br>3) Mouse Ultrasensitive Insulin ELISA Kit, Alpco, 80-INSMSU-E01, 09137<br>4) Human Serum Albumin Monoclonal Antibody (15C7), Invitrogen, MA1-90420, YE3901934<br>5) Human Serum Albumin Monoclonal Antibody (15C7), Abcam, Ab10241, GR3197685-10<br>6) Ultra Sensitive Mouse Insulin ELISA kit, Crystal Chem, 90080<br><br>Antibodies produced in-house used in experimental analysis:<br>- NIP-specific human IgG1 with M252Y/S254T/T256E/H433K/N434F (MST/HN) amino acid substitutions (doi: 10.4049/jimmunol.1401218)
Validation	All antibodies (commercial and in-house) are widely used in the laboratory and are regularly tested against in-house samples of known effect. Any abnormal results are detected, tracked, and reported (to manufacturer if necessary). In addition, the CoA of commercial antibodies were checked upon delivery.<br><br>The antibody produced in-house was validated by size-exclusion chromatography, SDS-PAGE, concentration measurements and ELISAs (antigen capture to confirm functional antigen binding).<br><br>1) Polyclonal anti-human albumin from goat, Sigma-Aldrich, A1151-1VL,<br>Selected citations: PMID: 29434196, PMID: 32816577<br>Citeab citation: https://www.citeab.com/antibodies/1487093-a1151-anti-albumin-antibody-produced-in-goat?des=2c46ce249af20431<br><br>2) ALP-conjugated anti-human albumin from goat, Bethyl Laboratories, Inc., A80-229AP<br>Selected citations: PMID: 25295540, PMID: 33445753<br>Citeab citation: https://www.citeab.com/antibodies/15655122-a80-229a-goat-anti-human-albumin-cross-adsorbed-ant?

des=fc6b203d53a0835e.

3) Mouse Ultrasensitive Insulin ELISA Kit, Alpco, 80-INSMSU-E01
Selected citations: PMID: 37216093, PMID: 37655336, PMID: 37766791, PMID: 37580307,PMID: 38068904.
Citeab citation: https://www.citeab.com/kits/10321297-80-insmsu-e01-mouse-ultrasensitive-insulin-elisa?des=50e097b9459b932b

4) Human Serum Albumin Monoclonal Antibody (15C7), Invitrogen, MA1-90420
Citeab citation: https://www.citeab.com/antibodies/88978-ma1-90420-human-serum-albumin-monoclonal-antibody-15c?des=7db3dd77805abba1

5) Human Serum Albumin Monoclonal Antibody (15C7), Abcam, Ab10241
Citeab citation: https://www.citeab.com/antibodies/765814-ab10241-anti-human-serum-albumin-antibody-15c7?des=

6) Ultra Sensitive Mouse Insulin ELISA kit, Crystal Chem, 90080
Selected citations: PMID: 37380764, PMID: 37137910
Citeab citation: https://www.crystalchem.com/ultra-sensitive-mouse-insulin-elisa-kit.html

Eukaryotic cell lines

Policy information about cell lines and Sex and Gender in Research

Cell line source(s)	Cell line sources:<br>- High Five, Invitrogen, B85502.<br>- Expi293F, Thermo Fisher Scientific, A14527.<br>- HMEC-1-HA-FcRn-EGFP; Boston Children's Hospital, Harvard Medical School and Harvard Digestive Diseases Center, USA; generation of described in doi: 10.1091/mbc.E13-04-0174.<br>- GLP-1R reporter, Indigo Biosciences/Bionordika, IB33001
Authentication	Authentication:<br>- High Five: visual inspection of morphology, proliferation rate monitored, protein production rates of in-house standardized his-tagged FcRn monitored<br>- Expi293F; visual inspection of morphology, proliferation rate monitored, protein production rates of in-house standardized albumin and antibody variants monitored<br>- HMEC1-HA-FcRn-EGFP; visual inspection of morphology, proliferation rate monitored, FcRn expression validated by EGFP expression on FACS, cellular recycling of FcRn-negative proteins monitored<br>- The GLP-1R reporter cells were provided as a single-use reagent as part of an assay kit. The aliqoute of reporter cells were thawed and used in one single experiment, and were not maintained in extended culture.
Mycoplasma contamination	All cell lines tested negative for mycoplasma contamination.
Commonly misidentified lines<br>(See ICLAC register)	NA.

Animals and other research organisms

Policy information about studies involving animals; ARRIVE guidelines recommended for reporting animal research, and Sex and Gender in Research

Laboratory animals	Hemizygous or homozygous human FcRn Tg32 mice (B6.Cg-Fcgrttm1Dcr Tg(FCGRT)32Dcr/DcrJ). In hemizygous human FcRn Tg32 mice, groups of male mice at 7–8 weeks of age were used. In homozygous human FcRn Tg32 mice, groups of female and male mice at 8–16 weeks of age were used. The mice weighed 20-30 g and each group consisted of 4 to 6 mice.
Wild animals	The study did not involve wild animals.
Reporting on sex	The pharmacokinetic study in hemizygous FcRn Tg32 mice included males only, while that in homozygous FcRn Tg32 mice included a mix of males and females. Hence, the findings of the study relate to both males and females. Disaggregated data for sex was not collected.<br><br>The glucose tolerance test (GTT) evaluating the in vivo functionality of the GLP-1 albumin fusion proteins was performed in groups of all males (homozygous human FcRn Tg32 mice). Mouse sex may have an effect on the performance of a metabolic model, and it is therefore recommended to study metabolic processes in same-sex cohorts (PMID: 32110077).
Field-collected samples	The study did not involve field-collected samples.
Ethics oversight	The in vivo half-life study in hemizygous human FcRn Tg32 mice was performed at The Jackson Laboratory (JAX Services, Bar Harbor, ME, USA), which was approved by the Animal Care and Use Committee at The Jackson Laboratory. All other animal experiments were carried out at the Section of Comparative Medicine, Oslo University Hospital Rikshospitalet (Oslo, Norway) in accordance with the national guidelines and regulations, upon approval by the Norwegian Food Safety Authority (FOTS ID 23998).

Note that full information on the approval of the study protocol must also be provided in the manuscript.

Plants

Seed stocks	Not applicable, the study did not involve plants.
Novel plant genotypes	Not applicable, the study did not involve plants.
Authentication	Not applicable, the study did not involve plants.
